# Prognostic Factors and Clinical Outcomes in Critically Ill Patients with Hematological Malignancies in the Intensive Care Unit

**DOI:** 10.3390/jcm15103717

**Published:** 2026-05-12

**Authors:** Recep Civan Yüksel, Ahmet Safa Kaynar, Hatice Metin, Şahin Temel, Canan Baran Ünal, Zehranur Yılmaz, Gülşah Akyol, Kürşat Gündoğan, Murat Sungur

**Affiliations:** 1 Department of Internal Medicine, Division of Intensive Care, Faculty of Medicine, Erciyes University, 38039 Kayseri, Turkey; asafakaynar@gmail.com (A.S.K.); drsahintemel@gmail.com (Ş.T.); kgundogan@erciyes.edu.tr (K.G.); msungur@erciyes.edu.tr (M.S.); 2 Department of Intensive Care, Gaziantep City Hospital, 27100 Gaziantep, Turkey; haticeayan_059@hotmail.com; 3 Department of Intensive Care, Sivas Numune Hospital, 58040 Sivas, Turkey; cbaran@erciyes.edu.tr; 4 Department of Internal Medicine, Faculty of Medicine, Erciyes University, 38039 Kayseri, Turkey; akizehranur@gmail.com; 5 Department of Internal Medicine, Division of Hematology, Faculty of Medicine, Erciyes University, 38039 Kayseri, Turkey; drgakyol@gmail.com

**Keywords:** hematological malignancies, intensive care unit, prognostic factors, mortality, critical illness

## Abstract

**Background/Objectives:** Patients with hematologic malignancies represent a high-risk population requiring intensive care due to infections, organ failure, and treatment-related complications. Despite advances in oncologic therapies and intensive care management, mortality remains high. This study aimed to evaluate prognostic factors and clinical outcomes in critically ill patients with hematologic malignancies admitted to the intensive care unit (ICU). **Methods:** Adult patients (≥18 years) with hematologic malignancies who were admitted to a medical ICU and stayed for at least 48 h were retrospectively included. Demographic characteristics, laboratory parameters, and APACHE II, SOFA, EASIX, and HALP scores, as well as mortality and organ support requirements, were evaluated. **Results:** A total of 108 patients were included. The median age was 61 years (IQR: 49–70), and 61% were male. The 28-day mortality was 64.8%. Overall, 83.3% of patients required invasive mechanical ventilation for at least 24 h. The median ICU length of stay was 5 days (IQR: 3–10). Median APACHE II and SOFA scores were 22 (IQR: 16–28) and 9 (IQR: 6–11), respectively. In multivariate analysis, SOFA score (OR: 1.218, 95% CI: 1.022–1.451) and the highest BUN level during ICU stay (OR: 1.034, 95% CI: 1.008–1.060) were independently associated with intubation. Admission creatinine level was the only independent predictor of renal replacement therapy (OR: 1.948, 95% CI: 1.081–3.510). APACHE II score was the only variable independently associated with 28-day mortality (OR: 1.064, 95% CI: 1.002–1.129). **Conclusions:** APACHE II showed a modest but statistically significant association with 28-day mortality in this cohort. Intubation and RRT requirements were mainly associated with organ dysfunction severity and renal impairment. Larger multicenter studies are needed to validate these findings and to better define risk stratification in critically ill hematologic patients.

## 1. Introduction

Critically ill patients with hematological malignancies were long considered a group unlikely to benefit from intensive care because of high mortality rates and an expected poor prognosis, and were therefore viewed as a population in whom ICU resources might be consumed without benefit. Over time, however, important advances in intensive care practice, antimicrobial therapy, and hematological cancer treatment have markedly improved survival and clinical outcomes in this population. Large prospective cohort studies have shown that ICU admission can provide meaningful survival benefits in selected patients with hematological malignancies and that both short- and long-term outcomes can be acceptable [1]. Furthermore, international multicenter studies in immunocompromised patients with acute respiratory failure have shown that outcomes are increasingly related to the severity of the acute illness and organ dysfunction rather than to the mere presence of the underlying malignancy, and that ICU mortality in these patients may be comparable to that of other critically ill populations [2,3]. These findings have contributed to a shift in the clinical approach to ICU admission in patients with hematological malignancies and have highlighted the importance of early recognition of organ dysfunction and timely intensive care support.

Early recognition of organ dysfunction is particularly important in critically ill hematologic patients because clinical deterioration may occur rapidly in the context of sepsis, treatment-related toxicities, immunosuppression, bleeding, and multiorgan involvement. Timely identification of respiratory, renal, cardiovascular, or circulatory failure may facilitate earlier ICU referral, closer hemodynamic monitoring, prompt organ support, and more coordinated decision-making between intensivists and hematologists. In this setting, identifying practical predictors that are readily available at ICU admission or during early ICU stay may have direct implications for daily clinical management.

The factors determining prognosis in ICU patients with hematological malignancies have been increasingly well defined in recent years. Several studies have shown that mortality is related not only to features of the underlying malignancy but also closely to the severity of acute illness and organ dysfunction [1]. In particular, high SOFA or APACHE scores, septic shock, acute kidney injury, the need for invasive mechanical ventilation, and vasopressor use have been reported as major determinants of ICU mortality [4]. Poor performance status, a history of allogeneic hematopoietic stem cell transplantation, and acute respiratory failure have also been associated with adverse prognosis [1,5]. Nevertheless, more recent studies suggest that some classical risk factors, such as malignancy subtype or stem cell transplantation history, may have a limited role on their own in determining mortality and that outcomes are largely driven by acute disease severity [3,6].

Although many studies have demonstrated improved survival in ICU patients with hematological malignancies in recent years, identifying clinical and laboratory parameters that can predict prognosis during ICU stay remains an important area of research. There is substantial heterogeneity across healthcare systems, geographic regions, and centers. Real-world data from different settings are valuable for better understanding the clinical characteristics and outcomes of this patient group [3]. In addition, much of the available literature comes from Western Europe and North America, and data from different patient populations such as those in Türkiye remain limited [4]. Therefore, identifying factors that may predict prognosis in critically ill patients with hematological malignancies and evaluating the center-specific outcomes of these patients are of great importance. The aim of the present study was to investigate clinical and laboratory parameters that may predict prognosis during ICU stay in patients with hematological malignancies admitted to the ICU and to evaluate the clinical outcomes of this patient group in our center.

## 2. Materials and Methods

### 2.1. Study Design and Setting

This study was a retrospective observational cohort study conducted in the medical intensive care unit of a tertiary university hospital. Patients admitted to the intensive care unit between 1 January 2017 and 1 September 2022 with a diagnosis of hematological malignancy were retrospectively evaluated. Local ethics committee approval was obtained from the Erciyes University Clinical Research Ethics Committee on 14 September 2022 (decision no. 2022/613). Due to the retrospective design of the study, the requirement for informed consent was waived by the Ethics Committee. Patient confidentiality was protected, and identifying information such as names and surnames was not recorded.

### 2.2. Study Population

Patients with hematological malignancies admitted to the intensive care unit of a tertiary university hospital between 1 January 2017 and 1 September 2022 were included. Only patients aged 18 years or older who were followed in the ICU for at least 48 h were enrolled.

**Inclusion criteria:**

- Age ≥ 18 years
- Presence of a diagnosis of hematological malignancy
- ICU stay of at least 48 h

**Exclusion criteria:**

- Age < 18 years
- Pregnancy
- Presence of a concomitant solid malignancy
- Incomplete clinical or laboratory data

For patients with multiple ICU admissions, only the first ICU admission was included in the analysis.

### 2.3. Data Collection

Clinical and laboratory data were retrospectively obtained from the hospital electronic medical record system. The following variables were recorded:

- Demographic characteristics (age, sex)
- Underlying hematological malignancy diagnosis
- Reason for ICU admission
- Laboratory parameters obtained at ICU admission, including renal function tests, liver function tests, electrolyte levels, complete blood count, albumin, and LDH
- Severity and prognostic scores calculated from digital records and laboratory parameters: Acute Physiology and Chronic Health Evaluation II (APACHE II), Sequential Organ Failure Assessment (SOFA), Endothelial Activation and Stress Index (EASIX), and Hemoglobin, Albumin, Lymphocyte, Platelet (HALP) scores

-EASIX was calculated as lactate dehydrogenase (LDH, U/L) × creatinine (mg/dL)/platelet count (10^9^/L).

-HALP was calculated as hemoglobin (g/L) × albumin (g/L) × lymphocyte count (10^9^/L)/platelet count (10^9^/L).

- ICU and hospital lengths of stay
- ICU and hospital mortality

Detailed hematologic disease characteristics such as remission/progression status, previous chemotherapy exposure, stem cell transplantation history, and baseline performance status were not consistently available in all patients because of the retrospective design and therefore could not be included in the final analyses.

### 2.4. Outcomes

The primary outcome of the study was 28-day mortality. Secondary outcomes included intubation requirement and renal replacement therapy requirement. Clinical and laboratory variables associated with these outcomes were also evaluated. Because the primary endpoint was predefined as a binary 28-day outcome, the main analytical framework was based on binary outcome models rather than time-to-event survival analysis.

### 2.5. Statistical Analysis

Statistical analyses were performed using IBM SPSS Statistics version 22.0. Continuous variables were expressed as median and interquartile range (IQR), and categorical variables as frequency and percentage (%). Mann–Whitney U test was used for comparisons between two independent groups, whereas Kruskal–Wallis test was used for comparisons among more than two groups. Wilcoxon signed-rank test was applied for dependent-group comparisons. Categorical variables were compared using the chi-square test or Fisher’s exact test when appropriate. Relationships between variables were evaluated using Spearman’s rank correlation coefficient. Variables entered into multivariable models were selected based on a combination of clinical relevance, prior literature, and results of univariate analyses, while also taking into account the relatively limited sample size and the need to reduce the risk of overfitting. Because composite severity scores and several laboratory parameters may reflect overlapping aspects of acute illness severity, potentially collinear variables were not forced into the same model unless considered clinically necessary. No formal a priori sample size calculation was performed because of the retrospective design of the study. A *p* value < 0.05 was considered statistically significant in all analyses.

## 3. Results

**Figure 1:**
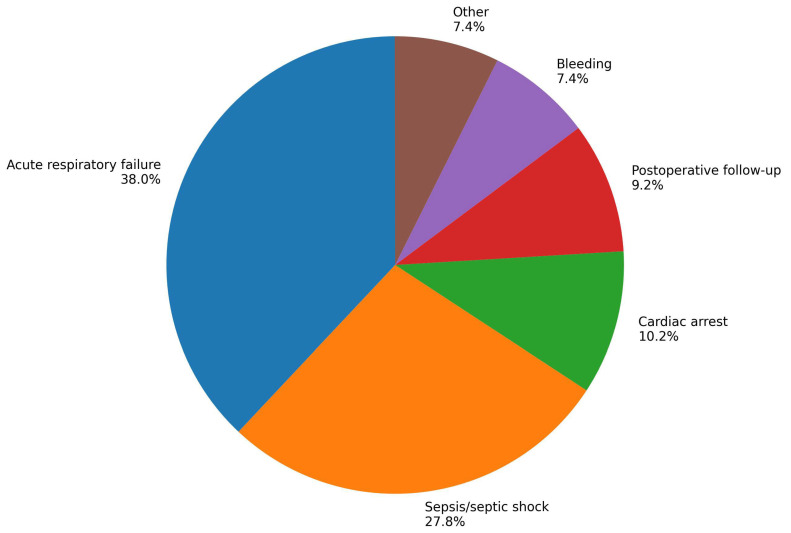
Distribution of ICU admission reasons.

A total of 108 patients with hematological malignancies admitted to the intensive care unit were included in the study. The median age was 61.0 years (IQR: 49.0–70.8), and 66 patients (61.1%) were male. Overall, 90 patients (83.3%) required invasive mechanical ventilation. The most common reasons for ICU admission were acute respiratory failure in 41 patients (38.0%) and sepsis/septic shock in 30 patients (27.8%), followed by cardiac arrest in 11 patients (10.2%), postoperative follow-up in 10 patients (9.3%), bleeding in 8 patients (7.4%), and other causes in 8 patients (7.4%) (Figure 1). The median SOFA score was 9.0 (IQR: 6.0–11.0), and the median APACHE II score was 22.0 (IQR: 16.0–28.0). The median ICU length of stay was 5.0 days (IQR: 3.0–10.0), and the median hospital length of stay was 21.0 days (IQR: 8.2–37.8). The median EASIX and HALP scores were 15.7 (IQR: 4.9–36.2) and 31.1 (IQR: 6.2–84.4), respectively. The 28-day mortality was 64.8% (*n* = 70) (Table 1).

In univariate analyses based on categorized laboratory variables, low platelet levels and high BUN, creatinine, INR, potassium, and sodium values were significantly associated with 28-day mortality. In contrast, albumin, age, white blood cell count, neutrophil count, lymphocyte count, hemoglobin level, AST, ALT, ALP, GGT, LDH, uric acid, calcium, phosphorus, EASIX score, and HALP score were not significantly associated with 28-day mortality (Table 2). In addition, admission creatinine and BUN showed borderline associations with 28-day mortality in analyses using continuous variables, suggesting a possible clinically relevant contribution despite the limited statistical power of the study. Although several clinical and laboratory parameters were associated with 28-day mortality in univariate analyses, only APACHE II remained independently associated with mortality in multivariable logistic regression. In the model including INR, creatinine, and sodium, each 1-point increase in APACHE II score increased the odds of 28-day mortality (OR: 1.064, 95% CI: 1.002–1.129, *p* = 0.043). In the same model, INR (OR: 0.844, 95% CI: 0.529–1.347, *p* = 0.478), creatinine (OR: 1.317, 95% CI: 0.812–2.135, *p* = 0.264), and sodium (OR: 1.035, 95% CI: 0.976–1.097, *p* = 0.254) were not independently significant. Model fit was acceptable according to the Hosmer–Lemeshow test (*p* = 0.516). Similarly, in an alternative model including BUN, INR, APACHE II, and platelet count, only APACHE II remained an independent predictor of mortality (OR: 1.063, 95% CI: 1.001–1.128, *p* = 0.045) (Table 3).

Patients who required intubation had significantly lower neutrophil counts (*p* = 0.005) and lower lymphocyte counts (*p* = 0.019). Low platelet levels (*p* = 0.032) and high BUN levels (*p* = 0.001) were also associated with intubation requirement. In addition, higher SOFA (*p* = 0.006) and APACHE II scores (*p* = 0.006) were observed in intubated patients. In contrast, albumin, age, hemoglobin, AST, ALT, ALP, GGT, LDH, creatinine, uric acid, sodium, potassium, calcium, phosphorus, EASIX score, and HALP score were not significantly associated with intubation requirement. In multivariable logistic regression analysis, SOFA score (OR: 1.218, 95% CI: 1.022–1.451, *p* = 0.028) and the highest BUN value measured during ICU stay (OR: 1.034, 95% CI: 1.008–1.060, *p* = 0.009) were identified as independent predictors of intubation requirement. The lowest neutrophil and platelet values measured during ICU stay were not significant in multivariable analysis. Model fit was acceptable (Hosmer–Lemeshow *p* = 0.064). In an alternative multivariable model including APACHE II score, the highest creatinine value and the lowest platelet and lymphocyte values during ICU stay, no independent significant predictor was identified; however, model fit remained acceptable (Hosmer–Lemeshow *p* = 0.437) (Table 4).

Patients who underwent renal replacement therapy had significantly higher BUN (*p* < 0.001), creatinine (*p* = 0.001), and uric acid levels (*p* = 0.005). High INR (*p* = 0.001), phosphorus (*p* = 0.001), and calcium (*p* = 0.028) were also associated with RRT requirement. In addition, a high APACHE II score (*p* = 0.027) and a high EASIX score (*p* = 0.038) were significantly associated with RRT requirement. In contrast, age, albumin, white blood cell count, neutrophil and lymphocyte counts, hemoglobin, platelet count, AST, ALT, ALP, GGT, LDH, sodium, potassium, and HALP score were not significantly associated with RRT requirement. In multivariable logistic regression analysis performed to identify independent predictors of RRT requirement, only creatinine at ICU admission remained an independent predictor. Higher creatinine levels were associated with increased odds of requiring RRT (OR: 1.948, 95% CI: 1.081–3.510, *p* = 0.026). In contrast, APACHE II score, highest INR value, and highest phosphorus value were not independently significant. Model fit was acceptable (Hosmer–Lemeshow *p* = 0.237), and the overall classification accuracy was 68.6%. In addition, no statistically significant association was found between RRT requirement and 28-day mortality (Pearson chi-square *p* = 0.759). In an alternative model including APACHE II, highest phosphorus, EASIX score, and lowest hemoglobin, low hemoglobin appeared significant; however, because model fit was inadequate, this model was not selected as the main analysis (Table 5).

## 4. Discussion

In this study, we evaluated the clinical characteristics, short-term outcomes, and prognosis-related parameters of ICU patients with hematological malignancies. The main findings were that several clinical and laboratory parameters appeared to be associated with 28-day mortality, but only APACHE II remained independently associated with mortality in multivariable analysis; the SOFA score and the highest BUN value measured during the ICU stay were independently associated with intubation requirement; and only creatinine level remained an independent predictor of RRT requirement in the main model. Overall, these findings suggest that prognosis in critically ill patients with hematological malignancies is closely related to the severity of acute physiologic deterioration and organ dysfunction rather than to the type of underlying malignancy alone.

The finding that APACHE II was the only variable independently associated with 28-day mortality is generally consistent with the existing literature. In the prospective multicenter study by Azoulay et al., the main determinants of prognosis were the severity of organ dysfunction, performance status, and the acute clinical presentation, whereas some classical malignancy-related features were not independently determinant [1]. In the retrospective Chinese cohort, APACHE II and SOFA scores were also associated with mortality, and invasive mechanical ventilation and renal dysfunction were related to poor outcomes [7]. Similarly, in a Turkish study of septic patients with hematological malignancies, the APACHE II score was identified as an independent mortality risk factor [8]. In our cohort, however, this association should be interpreted cautiously because the observed effect size was modest and the explanatory power of the final model was limited. Therefore, APACHE II should be regarded as a useful but not sufficient stand-alone prognostic marker in this patient population.

It is noteworthy that many laboratory abnormalities associated with mortality in univariate analysis lost significance in multivariable analysis. This may be explained in several ways. First, abnormalities such as elevated BUN, creatinine, electrolyte disturbances, and coagulation parameters may represent different reflections of the same acute illness burden, while composite scores such as APACHE II may already capture a substantial part of this physiologic deterioration. Second, laboratory abnormalities in ICU patients with hematological malignancies often occur in an interrelated manner, which may weaken their independent effects in multivariable models. Consistent with this, previous studies have suggested that factors such as neutropenia, transplant history, or malignancy subtype may not be as strong independent predictors as once thought, and that outcomes are more closely related to acute organ dysfunction [3,9].

Our findings regarding intubation requirement are also clinically meaningful. The identification of SOFA score as an independent predictor in multivariable analysis suggests that the need for intubation is primarily a reflection of multiple organ dysfunction and acute clinical deterioration. This is consistent with studies reporting that mechanical ventilation requirement is closely associated with poor prognosis in critically ill patients with hematological malignancies. In the study by Liu et al., invasive mechanical ventilation was associated with worse outcomes at all time points, and SOFA dynamics had strong prognostic value [7]. In the study by Kalicińska et al., acute respiratory failure was identified as one of the main variables associated with both ICU and hospital mortality [9]. In addition, recent reviews of critical illness in adults with hematological malignancies emphasize respiratory failure, sepsis, and acute kidney injury as the most common causes of ICU admission [10]. Therefore, the identification of SOFA as an independent predictor of intubation in our study is both expected and supported by the literature.

The identification of the highest BUN value measured during ICU stay as an independent predictor of intubation is also important. Elevated BUN may reflect renal dysfunction, but it may also serve as a marker of tissue hypoperfusion, catabolic burden, sepsis severity, and multiple organ failure. Because sepsis and acute kidney injury are frequent in patients with hematological malignancies, BUN may represent not only a nephrological marker but also an indicator of systemic disease severity. Current reviews and cohort studies have shown that AKI is both a common reason for ICU admission and a factor associated with poor outcomes in this patient group [9,10]. Our findings suggest that the need for intubation should be interpreted not solely as a pulmonary event but within a broader framework of multisystem deterioration. The lack of independent significance in the alternative model including APACHE II, highest creatinine, lowest platelet, and lowest lymphocyte values may indicate that prediction of intubation requirement is sensitive to the specific combination of variables used and that larger studies are needed.

Cardiovascular imaging may also be relevant in selected critically ill hematologic patients, particularly when pericardial involvement, intracardiac thrombi, or suspected cardiac masses are present. In this context, Paolisso et al. showed that several echocardiographic features—including infiltration, moderate-to-severe pericardial effusion, non-left localization, sessile implantation, polylobate shape, and inhomogeneity—may help identify malignant cardiac masses and guide the need for second-level imaging such as CMR, CCT, or PET [11]. Although cardiovascular imaging findings were not specifically analyzed in our cohort, a structured multimodality imaging approach may be useful when atypical intracardiac or pericardial findings are encountered in critically ill patients with hematologic diseases.

Although RRT requirement has been reported to be associated with mortality in the literature, no significant relationship was found between RRT and 28-day mortality in our study [9,12]. In multivariable analysis, only creatinine at ICU admission was identified as an independent predictor of RRT requirement. This discrepancy may be related to the limited sample size, patient heterogeneity, differences in clinical practice between centers, and the fact that RRT initiation may be influenced not only by disease severity but also by timing, clinician preference, and local treatment thresholds.

Although composite indices such as EASIX and HALP were evaluated in our study, they did not emerge as independent predictors in multivariable analyses. This may be related both to the limited sample size and to overlap between these indices and acute organ failure scores or basic biochemical parameters. Nevertheless, these indices may still provide prognostic value in selected subgroups or larger cohorts. Indeed, the continuing interest in novel biomarkers and risk stratification tools, including lactate dynamics or early warning scores, suggests that additional parameters may be useful in specific patient subsets [8]. Therefore, rather than excluding EASIX and HALP from prognostic assessment, we believe that their role should be re-evaluated in larger studies.

From a clinical perspective, our findings may contribute to daily ICU decision-making in hematologic patients. Severity scores such as APACHE II and SOFA may support early risk stratification, while recognition of worsening renal function or rising BUN levels may prompt closer monitoring, earlier organ support planning, and more timely communication between intensivists and hematologists. Although these variables should not be used in isolation for ICU triage, they may facilitate a more structured multidisciplinary approach in this high-risk population.

Our study has several strengths. First, it reflects real-world data and includes detailed clinical and laboratory information from ICU patients with hematological malignancies. Second, in addition to mortality, separate models were developed for clinically relevant outcomes such as intubation and RRT requirement. Third, the study provides regional data from Türkiye and thus contributes to the limited literature from non-Western settings.

This study has several limitations. First, its retrospective single-center design may have introduced selection bias and limits generalizability. Second, the relatively small sample size and number of outcome events restricted model complexity and increased the risk of overfitting. Third, inclusion of only patients with an ICU stay of at least 48 h may have introduced survival bias by excluding very early deaths after ICU admission. Fourth, detailed hematologic variables such as remission status, progression, prior chemotherapy exposure, stem cell transplantation history, and baseline performance status were not consistently available and therefore could not be analyzed. Fifth, ICU admission criteria and treatment strategies such as ventilation and sepsis management were not protocolized for the purpose of this study. Finally, because the primary endpoint was analyzed as a binary 28-day outcome, time-to-event analyses were not included, and survival dynamics over time could not be fully explored.

In conclusion, APACHE II was the only variable independently associated with 28-day mortality in this cohort, although the strength of this association was modest. The SOFA score and the highest BUN value were independently associated with intubation requirement, whereas admission creatinine was associated with RRT requirement. These findings support the central role of acute physiologic deterioration and organ dysfunction in critically ill patients with hematological malignancies. Future multicenter studies with larger sample sizes and more detailed hematologic data are needed to validate these findings and refine risk stratification in this population.

## Figures and Tables

**Table 1:** Baseline characteristics of the overall cohort.

Variable	Overall Cohort, Median (IQR) or *n* (%)
Age, years	61.0 (49.0–70.8)
Male sex, *n* (%)	66 (61.1)
Invasive mechanical ventilation, *n* (%)	90 (83.3)
SOFA score	9.0 (6.0–11.0)
APACHE II score	22.0 (16.0–28.0)
Albumin, g/dL	2.9 (2.7–3.5)
White blood cell count, /mm^3^	3945.0 (722.5–10,827.5)
Neutrophil count, /mm^3^	2355.0 (385.0–7147.5)
Lymphocyte count, /mm^3^	590.0 (80.0–1430.0)
Hemoglobin, g/dL	8.7 (7.5–9.7)
Platelet count, /mm^3^	43,500.0 (19,000.0–106,500.0)
LDH, U/L	389.0 (231.0–734.0)
BUN, mg/dL	36.0 (19.4–51.8)
Creatinine, mg/dL	1.2 (0.9–1.9)
INR	1.3 (1.2–1.5)
Potassium, mmol/L	4.0 (3.4–4.6)
Sodium, mmol/L	140.0 (136.0–144.0)
ICU length of stay, days	5.0 (3.0–10.0)
Hospital length of stay, days	21.0 (8.2–37.8)
EASIX score	15.7 (4.9–36.2)
HALP score	31.1 (6.2–84.4)
28-day mortality, *n* (%)	70 (64.8)

Abbreviations: IQR, interquartile range; SOFA, Sequential Organ Failure Assessment; APACHE II, Acute Physiology and Chronic Health Evaluation II; LDH, lactate dehydrogenase; BUN, blood urea nitrogen; INR, International Normalized Ratio; EASIX, Endothelial Activation and Stress Index; HALP, Hemoglobin, Albumin, Lymphocyte, Platelet score.

**Table 2:** Comparison of baseline variables according to 28-day mortality.

Variable	28-Day Non-Survivors (*n* = 70) Median (IQR)	28-Day Survivors (*n* = 38) Median (IQR)	*p*
Age, years	61.0 (49.75–68.50)	61.0 (46.25–72.25)	0.535
Albumin, g/dL	3.0750 (2.7475–3.5450)	2.8450 (2.6000–3.2550)	0.151
WBC, /mm^3^	3640 (565–12,295)	4640 (842.5–9075)	0.688
Neutrophil count, /mm^3^	2365 (329.5–8697.5)	2040 (667.5–5535)	0.737
Lymphocyte count, /mm^3^	540 (65–1900)	630 (80–1167.5)	0.970
Hemoglobin, g/dL	8.80 (7.50–9.725)	7.95 (7.35–9.70)	0.425
Platelet count, /mm^3^	39,500 (16,500–98,000)	51,000 (23,500–107,250)	0.429
LDH, U/L	375 (250.5–820)	410 (218–577.25)	0.917
BUN, mg/dL	37.95 (24.08–54.60)	27.25 (17.15–50.40)	0.054
Creatinine, mg/dL	1.3000 (0.9350–2.0050)	1.0550 (0.8025–1.5225)	0.050
INR	1.2600 (1.1950–1.5450)	1.2000 (1.1000–1.4800)	0.090
Potassium, mmol/L	3.9600 (3.2775–4.6625)	3.9950 (3.5700–4.4825)	0.755
Sodium, mmol/L	140.0 (135.75–144.25)	140.0 (136.75–143.00)	0.671
SOFA score	9.0 (6.0–11.0)	9.0 (5.75–11.0)	0.581
APACHE II score	23.0 (17.0–28.25)	19.0 (14.0–22.25)	0.023
ICU length of stay, days	6.5 (3.0–12.25)	5.0 (2.0–9.0)	0.200
Hospital length of stay, days	21.0 (10.5–34.5)	24.5 (7.5–46.25)	0.956
EASIX score	17.4754 (4.6953–43.9940)	11.9739 (4.9204–26.6608)	0.174
HALP score	34.6045 (6.4390–88.5175)	30.2050 (4.9414–66.1885)	0.569

Data are presented as median (interquartile range, IQR). *p* values were obtained using the Mann–Whitney U test.

**Table 3:** Multivariate logistic regression analysis for 28-day mortality. Dependent variable: 28-day mortality (0 = survivor, 1 = non-survivor).

Variable	B	SE	OR (Exp(B))	95% CI	*p*
INR	−0.169	0.238	0.844	0.529–1.347	0.478
APACHE II	0.062	0.030	1.064	1.002–1.129	0.043
Creatinine	0.275	0.247	1.317	0.812–2.135	0.264
Sodium	0.034	0.030	1.035	0.976–1.097	0.254

Abbreviations: CI, confidence interval; OR, odds ratio; SE, standard error. Model fit: Hosmer–Lemeshow *p* = 0.516; −2 Log likelihood = 131.319; Cox & Snell R^2^ = 0.071; Nagelkerke R^2^ = 0.098.

**Table 4:** Multivariate logistic regression analysis for intubation requirement.

Variable	B	SE	OR (Exp(B))	95% CI	*p*
SOFA	0.197	0.089	1.218	1.022–1.451	0.028
Peak BUN during ICU stay	0.033	0.013	1.034	1.008–1.060	0.009
Lowest neutrophil count during ICU stay	0.000	0.000	1.000	1.000–1.000	0.727
Lowest platelet count during ICU stay	0.000	0.000	1.000	1.000–1.000	0.806

Abbreviations: SE, standard error; CI, confidence interval; ICU, intensive care unit. Note: Dependent variable was intubation status (0 = not intubated, 1 = intubated). Highest BUN value during ICU stay, lowest neutrophil count during ICU stay, and lowest platelet count during ICU stay were entered into the model as continuous variables. Model fit: Hosmer–Lemeshow *p* = 0.064; −2 Log likelihood = 77.875; Cox & Snell R^2^ = 0.165; Nagelkerke R^2^ = 0.277; overall classification accuracy = 85.2%.

**Table 5:** Multivariate logistic regression analysis for renal replacement therapy requirement.

Variable	B	SE	OR (Exp(B))	95% CI	*p*
APACHE II	0.033	0.032	1.033	0.970–1.101	0.313
Admission Creatinine	0.667	0.300	1.948	1.081–3.510	0.026
Peak INR	0.483	0.297	1.620	0.905–2.902	0.105
Peak phosphorus	0.166	0.094	1.180	0.982–1.418	0.077

Abbreviations: CI, confidence interval; OR, odds ratio; SE, standard error. Model fit: Hosmer–Lemeshow *p* = 0.237; −2 Log likelihood = 119.567; Cox & Snell R^2^ = 0.211; Nagelkerke R^2^ = 0.282; overall classification accuracy = 68.6%. Dependent variable: renal replacement therapy requirement (0 = no RRT, 1 = RRT).

## Data Availability

The data presented in this study are available on request from the corresponding author due to privacy and ethical restrictions.

## References

[1] Azoulay E., Mokart D., Pène F., Lambert J., Kouatchet A., Mayaux J., Vincent F., Nyunga M., Bruneel F., Laisne L.-M. (2013). Outcomes of critically ill patients with hematologic malignancies: Prospective multicenter data from France and Belgium—A groupe de recherche respiratoire en réanimation onco-hématologique study. J. Clin. Oncol..

[2] Mehta S., Azoulay E., Munshi L., Demoule A., Perner A., Meyhoff T.S., Bauer P.R., Metaxa V., Pène F., Girault C. (2025). Sex-based differences in ICU management and outcomes of immunocompromised patients: A post hoc analysis of the prospective multicenter multinational Efraim cohort. J. Crit. Care.

[3] Munshi L., Darmon M., Soares M., Pickkers P., Bauer P., Meert A.-P., Martin-Loeches I., Staudinger T., Pene F., Antonelli M. (2021). Acute Respiratory Failure Outcomes in Patients with Hematologic Malignancies and Hematopoietic Cell Transplant: A Secondary Analysis of the EFRAIM Study. Transplant. Cell Ther..

[4] Bikmaz Ş.G.A., Gökçe O., Haşimoğlu M.M., Boyaci N., Türkoğlu M., Yegin Z.A., Özkurt Z.N., Yağci A.M. (2023). Risk factors for ICU mortality in patients with hematological malignancies: A single-center, retrospective cohort study from Turkey. Turk. J. Med. Sci..

[5] Lemiale V., Resche-Rigon M., Mokart D., Pène F., Rabbat A., Kouatchet A., Vincent F., Bruneel F., Nyunga M., Lebert C. (2015). Acute respiratory failure in patients with hematological malignancies: Outcomes according to initial ventilation strategy. A groupe de recherche respiratoire en réanimation onco-hématologique (Grrr-OH) study. Ann. Intensive Care.

[6] Lemiale V., Pons S., Mirouse A., Tudesq J.-J., Hourmant Y., Mokart D., Pène F., Kouatchet A., Mayaux J., Nyunga M. (2020). Sepsis and Septic Shock in Patients with Malignancies: A Groupe de Recherche Respiratoire en Réanimation Onco-Hématologique Study. Crit. Care Med..

[7] Liu J., Cheng Q., Yang Q., Li X., Shen X., Zhang L., Liu Z., Khoshnood K. (2015). Prognosis-related factors in intensive care unit (ICU) patients with hematological malignancies: A retrospective cohort analysis in a Chinese population. Hematology.

[8] Inci K., Aygencel G., Gökçe O., Türkoğlu M., Kaynar L.A., Can F., Yeğin Z.A., Özkurt Z.N., Yağcı A.M. (2024). Prognostic value of hyperlactatemia and lactate clearance in septic patients with hematological malignancies. Ann. Hematol..

[9] Kalicińska E., Kuszczak B., Dębski J., Szukalski Ł., Wątek M., Strzała J., Rybka J., Czyż J., Lech-Marańda E., Zaucha J. (2021). Hematological malignancies in Polish population: What are the predictors of outcome in patients admitted to Intensive Care Unit?. Support. Care Cancer.

[10] Spring J., Munshi L. (2022). Hematology Emergencies in Adults with Critical Illness: Malignant Hematology. Chest.

[11] Paolisso P., Foà A., Bergamaschi L., Graziosi M., Rinaldi A., Magnani I., Angeli F., Stefanizzi A., Armillotta M., Sansonetti A. (2023). Echocardiographic Markers in the Diagnosis of Cardiac Masses. J. Am. Soc. Echocardiogr..

[12] Ileri I., Coskun R., Temel S., Gundogan K., Sungur M. (2020). Evaluation of National Early Warning System for Mortality in Hematological Malignancy Patients Admitted to Intensive Care Unit: Prospective, Single Center, Observational Study. J. Crit. Intensive Care.

